# Severe anaphylactic reaction to cisatracurium during anesthesia with cross-reactivity to atracurium

**DOI:** 10.1515/med-2020-0126

**Published:** 2020-05-15

**Authors:** Kotryna Linauskienė, Gintarė Grincevičienė, Laura Malinauskienė, Audra Blažienė, Anželika Chomičienė

**Affiliations:** Vilnius University, Faculty of Medicine, Institute of Clinical, Medicine, Department of Chest diseases, Immunology and Allergology, Vilnius, Lithuania. Santariskiu 2, Vilnius LT-08661, Vilnius, Lithuania; Outpatient department, Republican Panevezys Hospital Smėlynės g. 25, LT-35144, Panevėžys, Lithuania; Vilnius University, Faculty of Medicine, Institute of Clinical Medicine, Department of Chest diseases, Immunology and Allergology, Vilnius, Lithuania. Santariskiu 2, Vilnius LT-08661, Vilnius, Lithuania; Vilnius University, Faculty of Medicine, Institute of Clinical Medicine, Department of Chest diseases, Immunology and Allergology, Lithuania. Santariskiu 2, Vilnius LT-08661, Vilnius, Lithuania; Vilnius University, Faculty of Medicine, Institute of Clinical Medicine, Department of Chest diseases, Immunology and Allergology, Vilnius, Lithuania. Santariskiu 2, Vilnius LT-08661, Vilnius, Lithuania

## Abstract

A case of a severe anaphylactic reaction during general anesthesia was reported. Despite the high suspicion for cefuroxime as a trigger of the anaphylactic reaction, cisatracurium emerged being the culprit drug. This case illustrates the importance of testing all drugs used during procedures/operations, trying to find a culprit of anaphylaxis.

## 1 Introduction

Anaphylaxis is a life-threatening condition. Drugs were the most common cause (58%) of 2,458 anaphylaxis-related deaths in the United States from 1999 to 2010. The incidence of an immediate hypersensitivity reaction during anesthesia was between 1/10,000 and 1/20,000 [1].

Neuromuscular blocking agents (NMBAs) are responsible for 63% of immediate allergic reactions during anesthesia, while latex, hypnotics, antibiotics, plasma substitutes, and morphine-like substances account for 14%, 7%, 6%, 3%, and 2% of reactions, respectively [2]. According to the French database over the period 2000–2011, the mortality rate from an immediate hypersensitivity reaction to NMBA administration was 4.1% [3].

## 2 Case report

A 40-year-old female patient (weight 55 kg) was scheduled to undergo laparoscopic ovarian cystectomy under general anesthesia. In the past, she underwent ovarian cystectomy and appendectomy under general anesthesia without any adverse reactions. Her clinical and laboratory examinations were normal. There was no history of allergy.

In the operating theater, the patient received 2 mg of midazolam and 100 µg of fentanyl both intravenously (i/v). General anesthesia was induced with propofol 200 mg i/v and cisatracurium 5 mg i/v. Five minutes later, 1.5 g of cefuroxime i/v was administered for the infection prophylaxis. Within a few minutes after the cefuroxime injection, face flushing and redness of the eyes were observed. Her heart rate rapidly increased to 135 bpm and BP was 39/29 mm Hg. The anaphylactic reaction was suspected. The patient was intubated and treated as anaphylactic shock until vital signs recovered. When vital signs were stabilized, the residual neuromuscular blockade was reversed by 2 mg of neostigmine. The operation was postponed.

Anaphylaxis to cefuroxime was suspected. After 4 weeks, skin tests with amoxicillin, ampicillin, benzyl penicillin, and cefuroxime were performed according to the ENDA requirements. Skin tests were negative. Then, an oral provocation test with cefuroxime was performed. It was also negative. Later, the patient was tested with other perioperative drugs cisatracurium, fentanyl, midazolam, and propofol. Skin prick and intradermal tests were performed with the nonirritative concentrations of the drugs (Table 1) [1]. The skin prick test wheal larger than 3 mm accompanied by erythema compared with a negative response to the control saline solution was considered as positive. The intradermal test was considered positive when the size of the initial wheal increased by at least 3 mm in diameter after 20 min or increased twice that of the post-injection wheal. Late reading was performed for the following 24 h. Histamine was used as a positive control while a saline solution was used as a negative control.

**Table 1:** Concentrations of drugs used for skin prick and intradermal tests

	Prick	Intradermal
a. Concentrations of beta-lactams used to skin prick and intradermal tests.		
Benzylpenicillin	20,000 U/mL	20,000 U/mL
Ampicillin	20 mg/mL	20 mg/mL
Amoxicillin	20 mg/mL	20 mg/mL
Cefuroxim	2 mg/mL	2 mg/mL

b. Concentrations of general anaesthetic agents used for skin prick and intradermal tests.		
Atracurium	1 mg/mL	0.01 mg/mL*
Cis-atracurium	2 mg/mL	0.02 mg/mL*
Rocuronium	10 mg/mL	0.05 mg/mL
Suxamethonium	10 mg/mL	0.1 mg/mL
Midazolam	5 mg/mL	0.5 mg/mL
Fentanyl	0.05 mg/mL	0.005 mg/mL
Propofol	10 mg/mL	1 mg/mL

^*^Intradermal tests with cisatracurium and atracurium were not performed, because skin prick tests were positive.

**Figure 1:**
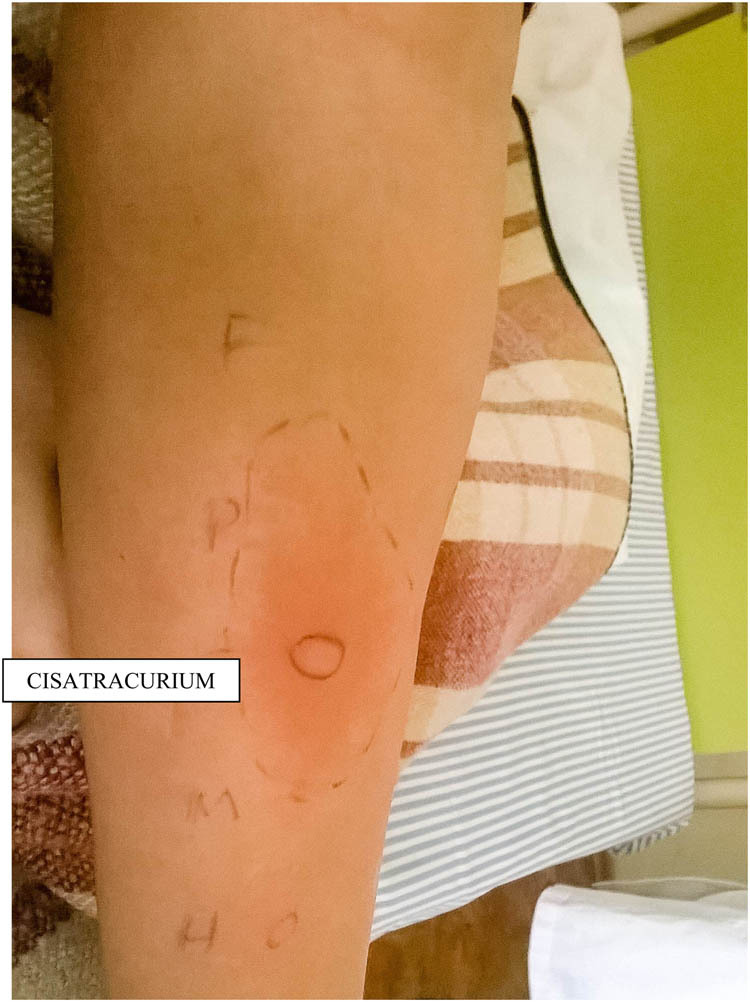
Positive skin prick test to cisatracurium. The diameter of a wheal is 9 mm, erythema – 55 mm.

**Figure 2:**
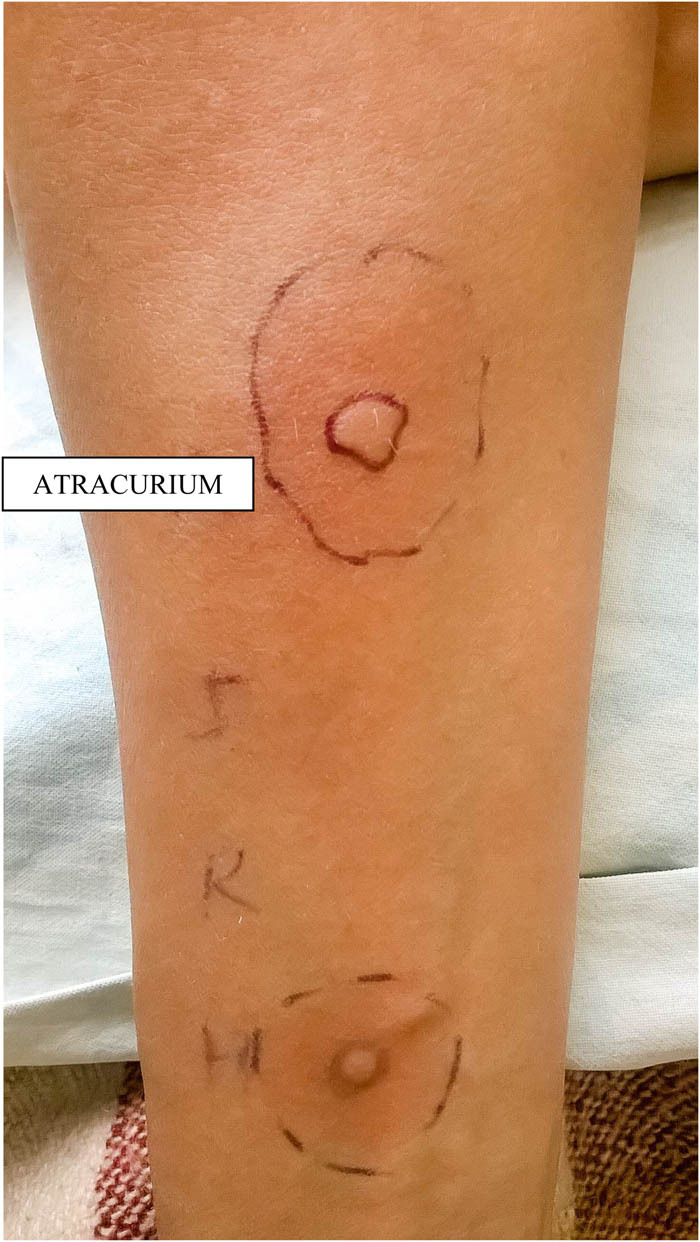
Positive skin prick test to atracurium due to cross-reactivity. The diameter of a wheal and erythema was 8 mm, erythema – 28 mm.

Skin tests with cisatracurium and other NMBAs (atracurium, rocuronium, and suxamethonium) were performed due to existing cross-reactivity between them (Table 1). Skin prick tests with cisatracurium (Figure 1) and atracurium (Figure 2) were positive. Cisatracurium was identified as the cause of anaphylaxis. Skin tests with other drugs used and latex were negative.

The consent for case report publication and pictureusage was obtained from the patient.

## 3 Discussion

Some patients with NMBA IgE-mediated anaphylaxis have never been exposed to NMBAs before. In this case, the cisatracurium was used during previous ovarian cystectomy 2 years ago and that could lead to sensitization. NMBAs are divalent ammonium compounds containing tertiary and quaternary ammonium groups that are widespread in the human environment including cosmetics, disinfectants, and other drugs. The significant increase in IgE-dependent sensitization to NMBAs and quaternary ammonium ion compounds is noticed in hairdressers and pholcodine (opioid antitussive) consumers [4]. For example, in Norway, there has been observed a significant decrease of sensitization to NMBAs after the withdrawal of the antitussive pholcodine [5].

Up to 50% of adverse reactions to NMBAs are considered to result from direct nonspecific mast cell and basophil activation leading to histamine release [6].

Cross-sensitivity between different NMBAs is frequent and observed in 60–70% of patients allergic to NMBAs. Cross-reactivity is more frequent within amino steroid groups (rocuronium, pancuronium, vecuronium, pipecuronium, rapacuronium) than within benzylisoquinoline-derived NMBAs (tubocurarine, atracurium, cisatracurium, mivacurium, doxacurium) [7,8]. It is also known that benzylisoquinolines are more potent histamine releasers than aminosteroidal NMBAs and can cause life-threatening allergic reactions [9].

Anaphylactic reactions occur rapidly and commonly involve the skin (rashes, urticaria, flushing, erythema, angioedema), cardiovascular (tachycardia, hypotension), and respiratory (rhino conjunctivitis, bronchospasm) systems, but may involve any other system such as gastrointestinal, central nervous, and genitourinary. The difference between immune- and nonimmune-mediated reactions cannot be made on clinical symptoms, but usually, nonimmune mediated reactions are less severe [7,10].

Skin tests carried out 4–6 weeks after anaphylactic reaction, combined with history, are the base for an accurate diagnosis. Tests should be performed with all the drugs used during the anesthesia. The established sensitivity of the skin tests for NMBAs is 94–97% [6].

Skin tests before anesthesia are recommended just for the patients who are at risk of anaphylaxis, e.g., a: who had an unexplained anaphylactic reaction to an unidentified allergen during previous anesthesia; b: who is known to be allergic to drug classes that will be used during anesthesia; and c: who is at risk for latex allergy [6].

## 4 Conclusion

This case emphasizes the importance of testing all drugs used during the procedure which resulted in anaphylaxis.
